# Interpreting airborne pandemics spreading using fractal kinetics’ principles

**DOI:** 10.12688/f1000research.53196.1

**Published:** 2021-07-20

**Authors:** Panos Macheras, Athanasios A. Tsekouras, Pavlos Chryssafidis

**Affiliations:** 1 Faculty of Pharmacy, Laboratory of Biopharmaceutics Pharmacokinetics, National and Kapodistrian University of Athens, Athens, 11526, Greece; 2 Athena Research Center, Attica, Athens, 15125, Greece; 3 Department of Chemistry, Laboratory of Physical Chemistry, National and Kapodistrian University of Athens, Athens, 11526, Greece

**Keywords:** fractal kinetics, COVID-19, airborne pandemics, herd immunity policy, SI model

## Abstract

Introduction

The reaction between susceptible and infected subjects has been studied under the well-mixed hypothesis for almost a century. Here, we present a consistent analysis for a not well-mixed system using fractal kinetics’ principles.

Methods

We analyzed COVID-19 data to get insights on the disease spreading in absence/presence of preventive measures. We derived a three-parameter model and show that the “fractal” exponent h of time larger than unity can capture the impact of preventive measures affecting population mobility.

Results

The h=1 case, which is a power of time model, accurately describes the situation without such measures in line with a herd immunity policy. The pandemic spread in four model countries (France, Greece, Italy and Spain) for the first 10 months has gone through four stages: stages 1 and 3 with limited to no measures, stages 2 and 4 with varying lockdown conditions. For each stage and country two or three model parameters have been determined using appropriate fitting procedures. The fractal kinetics model was found to be more akin to real life.

Conclusion

Model predictions and their implications lead to the conclusion that the fractal kinetics model can be used as a prototype for the analysis of all contagious airborne pandemics.

## Introduction

Recently, Jewell *et al.* ^1^ criticized the predictive models of the COVID-19 pandemic. This rigorous analysis justifies the first portion of the famous quote by George Box ^2^ “All models are wrong, some of them are useful”. All epidemiological models used in practice have a common origin, namely, the famous Kermack–McKendrick model. ^3^ We argue in this work that their poor predictive power originates from the erroneous hypothesis of the “well-mixed” epidemiological system; this hypothesis is crucial for the validity of the differential equations, which describe the “reaction” between susceptible ( *S*) and infected ( *I*) subjects. We also argue that the violation of this hypothesis results in a wrong perception and definition of the basic reproductive number *R*_0_^4, 5^ of epidemiological models, which denotes the number of secondary infections produced by a single infection.

People worldwide are concerned about the uncontrolled “exponential” spread of a disease, yet it is not clear or justified if this description is correct. In fact, an alternative “power” model based on an adjustable exponent of time has been proposed. ^6^ We expand this approach by first questioning the ‘well-mixed” hypothesis and introducing a “fractal kinetics’” approach ^7^ which yields, as a special case, the “power” model. This model ^7^ relies on fractal kinetics’ principles that are suitable for the study of reactions and diffusion processes in insufficiently mixed media. ^8, 9^ In the same vein, we explored all theoretical aspects of the fractal kinetics’ *SI* model and applied it for the description of the time evolution of the COVID-19 pandemic in several countries. Our results support that this “conceptual change” from classical to fractal kinetics principles offers a novel, useful approach for the analysis of airborne pandemics data and justifies the second portion of George Box ^2^ quote above.

## Theory

*The “reaction” of susceptible-infected individuals under homogeneous conditions.*

In the Kermack–McKendrick model, ^3^ the studied population is divided into susceptible, *S*, infectious, *I* and recovered, *R,* sub-populations while the relevant terms *SI* and *SIR* model were coined a long time ago. For each one of the sub-populations, specific ordinary differential equations are written based on the principles of chemical kinetics. These equations rely on the law of mass action ^10^ which states that the rate of the chemical reaction is directly proportional to the product of concentrations of the reactants. However, this law applies under the strict hypothesis that the studied chemical reaction takes place under well-stirred conditions. This dogma applies well in chemical systems and validates the use of time-independent reaction rate constants and molar concentrations of the reactants in the reaction rate expressions. Obviously, the well-mixed hypothesis cannot be applied to epidemiological models since individuals, unlike molecules in a stirred solution, do not mix homogeneously; this is particularly so when preventive measures are applied. This, in turn, makes the mathematical formalism used so far questionable and the derived estimates of the relevant parameters, *e.g.*, *R*_0_, a very rough approximation of reality. In fact, *R*_0_ cannot capture time-dependent variations in the transmission potential; the time course of an epidemic can be partly described by the effective reproduction number, *R*( *t*), which is a time-dependent parameter defined ^11, 12^ as the actual average number of secondary cases per primary case at time *t*:

$$R\left(t\right)=\frac{S\left(t\right)}{S\left(0\right)}R_{0}$$ (1)

where *S*( *t*) and *S*( *0*) are the numbers of susceptible subjects at time *t* and zero, respectively. Eq. 1 shows that *R*( *t*) relies on an estimate of *R*_0_, which is usually derived from the early phase data of the pandemic. *R*_0_ is also crucial for the calculations of herd immunity. ^5^

The current *SIR* models for the ongoing COVID-19 epidemic include additional features to the classical *SIR* model, ^3, 5^ namely, the probability of death in the vulnerable fraction of the population, infectious period, and a time from infection to death are included. ^13, 14^ The basic reproduction number, *R*_0_, and all variables and parameters of the model are expressed as Gaussian distributions around previously estimated means. ^2, 4, 14^ However, *R*( *t*) is used extensively as a reliable measure of a pathogen’s transmissibility. ^15, 16^

*The “reaction” of susceptible-infected individuals under heterogeneous conditions.*

In 1988, Kopelman ^8^ introduced the concept of fractal reaction kinetics for reactions taking place under topological constraints. Under these heterogeneous conditions, time-dependent coefficients *k*( *t*) and not rate constants govern the rate of the reaction process. ^8^ Numerous disciplines ^9, 17– 28^ study rate processes with this approach. It is also very appropriate in studying the “reaction” of susceptible-infected individuals under “real-life” conditions.

**Figure 1.**
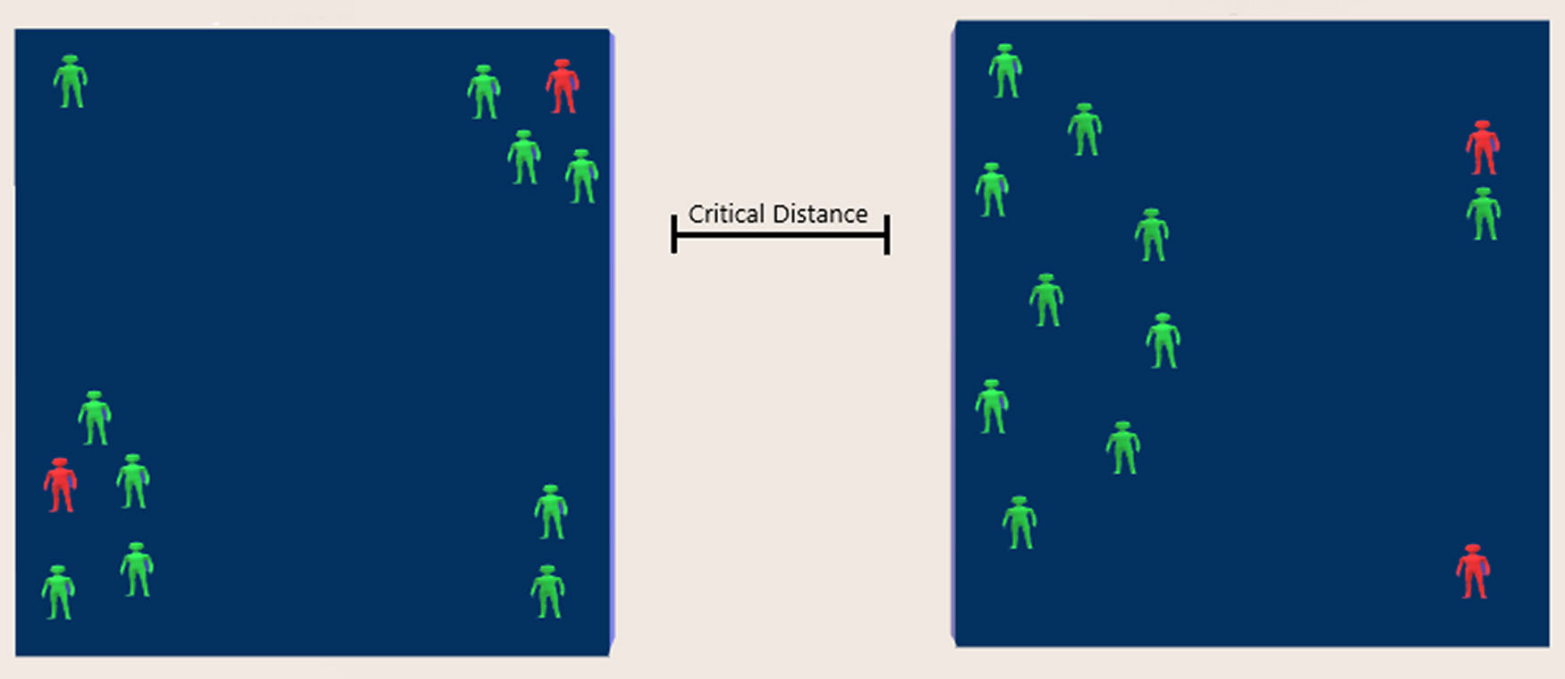
Probability considerations for virus transmission based on the “pair-up” and “critical distance” concepts of bimolecular reactions in fractal kinetics. ^8^ Although the number of infected (red) and susceptible subjects (green) is the same in both rooms, the instantaneous probability for virus transmission is 7/10 and 1/10 for the left- and right-hand side room, respectively.

Consider two rooms of the same size shown in Figure 1 with the same “concentration” of 10 unmovable susceptible subjects and two COVID-19 infected subjects. The probability for SARS-CoV-2 transmission is much higher in the left-hand side room, because the distance for seven of the susceptible subjects from the two infected subjects is much smaller than the “critical distance” associated with the bimolecular reactions of fractal kinetics. ^8^ On the contrary, only one of the susceptible subjects is within “critical distance” from infected subjects of the right-hand side room. The static picture depicts the equivalency of the social distancing (1.5 meters applied during the Covid-19 pandemic) with the “pair up” and “critical distance” concepts of fractal kinetics. ^8^ Intuitively, if the subjects in the two rooms start moving, virus transmission will increase as a function of time and will be dependent on the trajectory of each individual. Obviously, continuous movement of the subjects in the two rooms sweeping the available space would result in the transmission of the disease to all susceptible subjects in accordance with the “well-mixed” hypothesis. This means that the “well-mixed” system is just a single limiting case of the myriad heterogeneous space/time configurations of the individuals in a population.

These considerations lead us to the following very important conclusions relevant to airborne pandemics.

a. The time evolution of pandemics described by the classical *SI* and *SIR* models, ^5^ which are based on the well-mixed hypothesis, are very crude approximations of reality.
b. The use of a fixed *R*_0_ value, ^4, 5^ is inadequate for capturing the transmission dynamics. The use of *R*( *t*) can capture time-dependent variations in the transmission potential, ^11, 12, 15, 16^ but is heavily dependent on the *R*_0_ estimate. In real-life conditions, the transmission of the disease is not only dependent on time, but also on the topology/movement associated with susceptible/infected individuals.
c. The importance of the “initial conditions” for fractal reaction kinetics has been delineated. ^8^ In pandemics, the corresponding “initial conditions” are “patient zero” at the epicenter of the country of pathogen’s origin as well as “patient zeros” of the first humans infected in different countries. For the COVID-19 pandemic specifically, since most of the infected subjects are asymptomatic during the initial phase of the disease spreading, no precautions are taken. During this initial period, which lasts until social distancing measures are applied, disease spreading follows a “herd immunity” ^5^ style, which we call “herd kinetics”. Similarly, we coin the term “fractal kinetics” for the disease spreading when containment measures are imposed.

The fractal kinetics’ *SI* model ^7^ for epidemic spreading relies on the following equation:

$$\frac{\mathrm{dI}\left(t\right)}{\mathrm{dt}}=\frac{\beta }{t^{h}}I\left(t\right)\left[1-I\left(t\right)\right]$$ (2)

where *I*( *t*) is the cumulative fraction of infected individuals at time *t*, *β* is a parameter proportional to the probability of an infected individual to infect a healthy one and *h* is the fractal dimensionless exponent associated with fractal kinetics. ^8^ The core assumption of the model is that societies as complex systems will exhibit self-organization as a reaction to the emergence of a pandemic wave, enforcing preventive measures and increasing public awareness. Thus, instead of an infection rate constant, the fractal *SI* model uses a rate factor *β/t ^h^* decreasing of time. The solution of Eq. 2 gives *I*( *t*) as a function of time: ^7^

$$I\left(t\right)={\left[1+\text{cexp}\frac{\beta t^{1-h}}{h-1}\right]}^{-1}\;$$ (3)

where *c* is a parameter which determines the fraction of individuals that will become infected eventually.

By substituting $\beta =a^{1-h}$ we introduce parameter *α* of inverse time dimension, ^29^ which changes Eq. 3 into 4, namely:

$$I\left(t\right)={\left[1+\text{cexp}\frac{{\left(\mathrm{\alpha t}\right)}^{1-h}}{h-1}\right]}^{-1}$$ (4)

In the limit *t* → ∞ we find: ^7^

$$\lim_{t\to \infty }I\left(t\right)=\left\{\begin{array}{cc} 1, & h\leq 1\\ {\left(1+c\right)}^{-1}, & h>1 \end{array}\right\}$$ (5)

**Figure 2.**
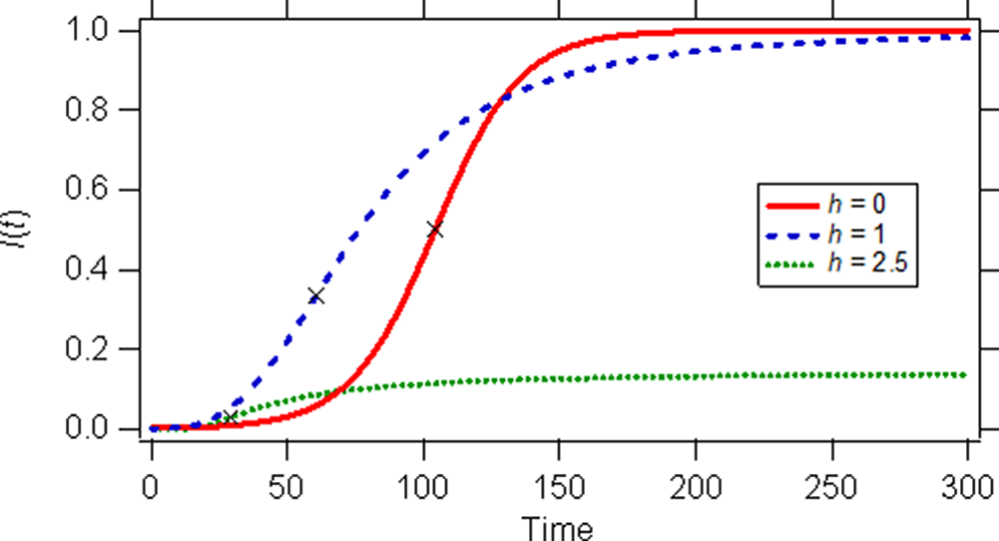
Simulated curves for the infected population fraction generated from Eqs. 4 and 6. Parameter values used: *h* = 0, *β* = 0.064, *c* = 790; *h* = 1, *β* = 3, *c* = 4.4 × 10 ^5^; *h* = 2.5, *α* = 0.018 (time) ^−1^, *c* = 6. Marks on the curves are inflection points.

The “well-mixed” model, described by Eq. 2 with *h* = 0, has a limiting value of *I*( *t*) equal to one as a result of a completely susceptible population. However, this is not a realistic feature for all pandemics that have appeared so far. Eq. 5 reveals that the plot of *I*( *t*) *versus* time for *h* > 1 reaches a plateau equal to 1/(1 + *c*) (see Figure 2), which is a reasonable feature for all pandemics. For the special case *h* = 1, Eq. 6 (also plotted in Figure 2) is derived which describes what we call “herd kinetics” not only because no precautions or measures are taken, but also because the rate of increase of infected subjects progressively diminishes in a similar fashion when a “herd immunity” ^5^ policy is implemented:

$$I\left(t\right)=\frac{1}{1+ct^{-\beta }}$$ (6)

A linearized form of Eq. 6 is as follows:

$$\ln\left(\frac{1}{I\left(t\right)}-1\right)=\mathrm{lnc}-\text{βlnt}$$ (7)

where the slope *β* is an “apparent” dimensionless transmissibility rate constant during the “herd kinetics” period; the term “apparent” is used to underline its proportional dependency to the probability of an infected individual to infect a healthy one (see Eq. 2). At *t* = 1, hence ln *t* = 0, we get:

$$I\left(t=1\right)=\frac{1}{1+c_{1}}$$ (8)

Theoretically, the value of *I*( *t* = 1) corresponds to the “initial conditions”, *i.e.*, the fraction of infected individuals at the first day of the pandemic; since the real “time zero” is unknown, *c*_1_ is proportional to the number of total (asymptomatic and symptomatic) infected cases from the real time zero to time *t* = 1 day, when the first case was confirmed. We use the notation *c*_1_ to distinguish it from *c* appearing in Eqs. 5, 6 and 7.

In all pandemics, a characteristic time is observed when the daily number of confirmed infected cases does not increase anymore and starts declining; this corresponds to the inflection point *t*_ip_. When *h* > 1, an estimate for *t*_ip_ can be obtained by equating the second derivative of Eq. 4 to zero and solving the resulting equation for time. Lacking an analytical solution, this equation can only be solved numerically.

For the special case *h* = 0, *t*_ip_, can be derived from Eq. 4:

$$t_{ip}=\frac{1}{\beta }\ln\left(c\right)$$ (9)

The following *t*_.ip_ can be derived from Eq. 2, under “herd kinetics” conditions ( *h* = 1):

$$t_{\mathrm{ip}}={\left(\frac{\left(\beta -1\right)c}{\beta +1}\right)}^{\frac{1}{\beta }}\text{for}\beta >1$$ (10)

The inflection points for the three examples considered, *h* = 0, *h* = 1 and *h* = 2.5 are shown on the simulated curves of Figure 2. Inflection points denote when a curve changes from being convex (upwards) to concave (downwards), *i.e.*, the confirmed infected new cases remain temporarily constant and then start to drop.

If the value of parameter *c* is low, all cases reach the asymptotic limit of 1. However, in real-life conditions the limiting value of the cumulative fraction of infected individuals, *I*( *t*) is always much smaller than 1. This epidemiological evidence (fact) can be explained only by the fractal kinetics *SI* model as shown in Figure 2. The curve of the example considered using *h* = 2.5 reaches the plateau value of 0.125, *i.e.*, 12.5% of the population will be infected eventually.

For *h* > 1, the *I*( *t*) corresponding to the inflection time point, *I* ( *t*_ip_) can be derived from Eq. 4 using the *t*_ip_ estimate in the denominator of Eq. 4. The *t*_ip_ estimate is obtained by equating the second derivative of Eq. 4 to zero and solving numerically the resulting equation.

For *h* = 0:

$$I{\left(t\right)}_{\mathrm{ip}}=0.5$$ (11)

while for *h* = 1:

$$I{\left(t\right)}_{\mathrm{ip}}=\frac{\beta -1}{2\beta }$$ (12)

During the time course of the pandemics, an estimate for the time of the termination or close to the termination of the spreading is desperately needed as early as possible. An estimate for the time of 90% termination, *t*_90%_ for *h* > 1, can be derived from Eq. 4 using *I*( *t*) = 0.90/(1 + *c*):

$$t_{90\%}=\frac{1}{\alpha }{\left[\left(h-1\right)\ln\left(\frac{1.1c+0.1}{c}\right)\right]}^{\frac{1}{1-h}}$$ (13)

## Methods

### Fits to COVID-19 data

The best fits of Eqs. 4 and 6 to the data ^30^ were obtained by maximizing the *R* ^2^ of the two adjacent periods. By anchoring the date of each country’s lockdown decision (or any similar form of draconian measure) and moving forward in time, the Levenberg–Marquardt algorithm of least squares was implemented. The lockdown dates are close or very close to the transition from herd kinetics to fractal kinetics and *vice versa.* A minimum value of *R* ^2^ = 0.985 was set as a criterion of goodness of fit and every value higher than that was accepted. The turning time data point at which the best *R* ^2^ value began to diminish was rejected and its prior time data point was accepted. From that time segment and further on the consequent kinetic profile was fitted to the data points until the plateau of quasi-steady state was reached. The fitting discontinuities observed in the kinetics between the distinct periods ( *e.g.*, from second to third period for France) are associated with the fact that *I*( *t*) values at the boundary of the two periods were not equalized in our fitting methodology. Between the quasi-steady state and the beginning of the second herd period a 10% change of the number of cumulative infected cases at one week interval was sought in order to establish the commencement of a second viral wave and the reproduction of the according fitting procedure. Data acquisition, modelling and simulations were programmatically implemented with Python language ^31^ and its respective libraries.

## Results

In our previous studies ^7, 32^ on COVID-19 data analysis, we applied the fractal kinetic *SI* model ( Eq. 3) assuming that fractal kinetics commences at time zero. However, reconsideration of the topological characteristics of the virus transmission in the light of Eq. 4 led us to the realization that a “herd kinetics’” period precedes the “fractal kinetics’” period. Exponent *β* drives the kinetics during the “herd kinetics” stage and is the analogue of *R*_0_ for a not well-mixed system. But, unlike *R*_0_, *β* is not associated with the expected number of cases directly generated by *one case* in a population. During the “fractal kinetics” period, parameter *α* in Eq. 4 governs the rate of the disease, while the prevailing spatial conditions are reflected on the *h* value. During this period, a meaningful parameter for the rate of the process is the half-life, *t*_½_ = ln2/ *α.*

### The “Herd-Fuzzy-Fractal-Herd-Fuzzy-Fractal” (HFF) ^2^ kinetic motif

**Figure 3.**
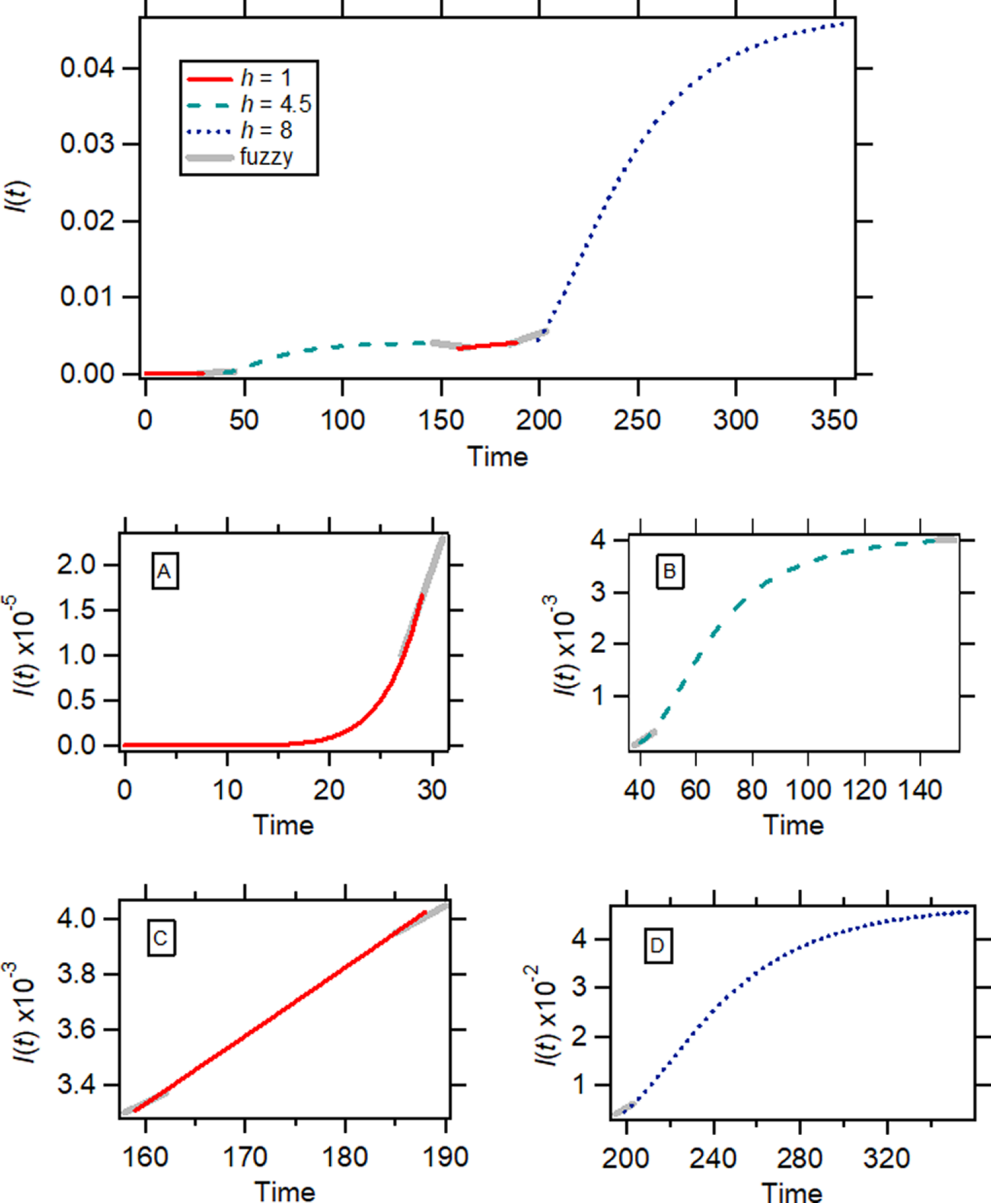
A schematic of the “Herd-Fuzzy-Fractal-Herd-Fuzzy-Fractal” (HFF) ^2^ kinetic motif of COVID-19 pandemic. The gray line segments indicate fuzzy periods. A–D. Subplots correspond to the four distinct periods of the kinetic motif. Equations and parameter values used: A: Eq. 6, *β* = 8, *c* = 3 × 10 ^16^; B: Eq. 4, *h* = 4.5, α = 0.012 (time) ^−1^, *c* = 240; C: Eq. 6, *β* = 1.18, *c* = 119350; D: Eq. 4, *h* = 8, *α* = 3.35 × 10 ^−3^(time) ^−1^, *c* = 20.

Initially, virus transmission takes place under “herd kinetics’” conditions ( Eq. 6, Figure 3A). This prevails until the first preventive measures are imposed; these can be followed by a lockdown decision. The preventive measures and the lockdown status induce a gradual reduction in the rate of the disease spread, *i.e.*, “fractal kinetics” starts operating ( Eq. 4, *h*>1, Figure 3B). The transition from herd kinetics ( Eq. 6) to fractal kinetics ( Eq. 4, *h*>1) can be gradual during this fuzzy period, with both equations operating concurrently. The prevalence of fractal kinetics during the lockdown period results in an asymptotic approach of *I*( *t*) to the steady state, *i.e.*, *I*( *t*) = (1+ *c*) ^-1^ (see Eq. 5, Figure 3B); according to Eq. 4 the higher the value of the fractal exponent of time *h*, the more rapid is the approach of *I*( *t*) to the steady state. This pattern we call “Herd-Fuzzy-Fractal” (HFF) kinetic motif. When the confirmed new cases reach a steady state, governments relax lockdown rules. In theory, when such a decision is taken, the termination of the first wave of the pandemic has been accomplished. However, the relaxation of lockdown measures in conjunction with the large number of infected individuals at steady state can, after a while, initiate a second wave of the pandemic leading to the application of new preventive measures and new lockdown rules. Consequently, a second wave of the disease emerges ( Figures 3C and 3D); hence, the (HFF) ^2^ kinetic motif.

### Analysis of COVID-19 data

**Figure 4.**
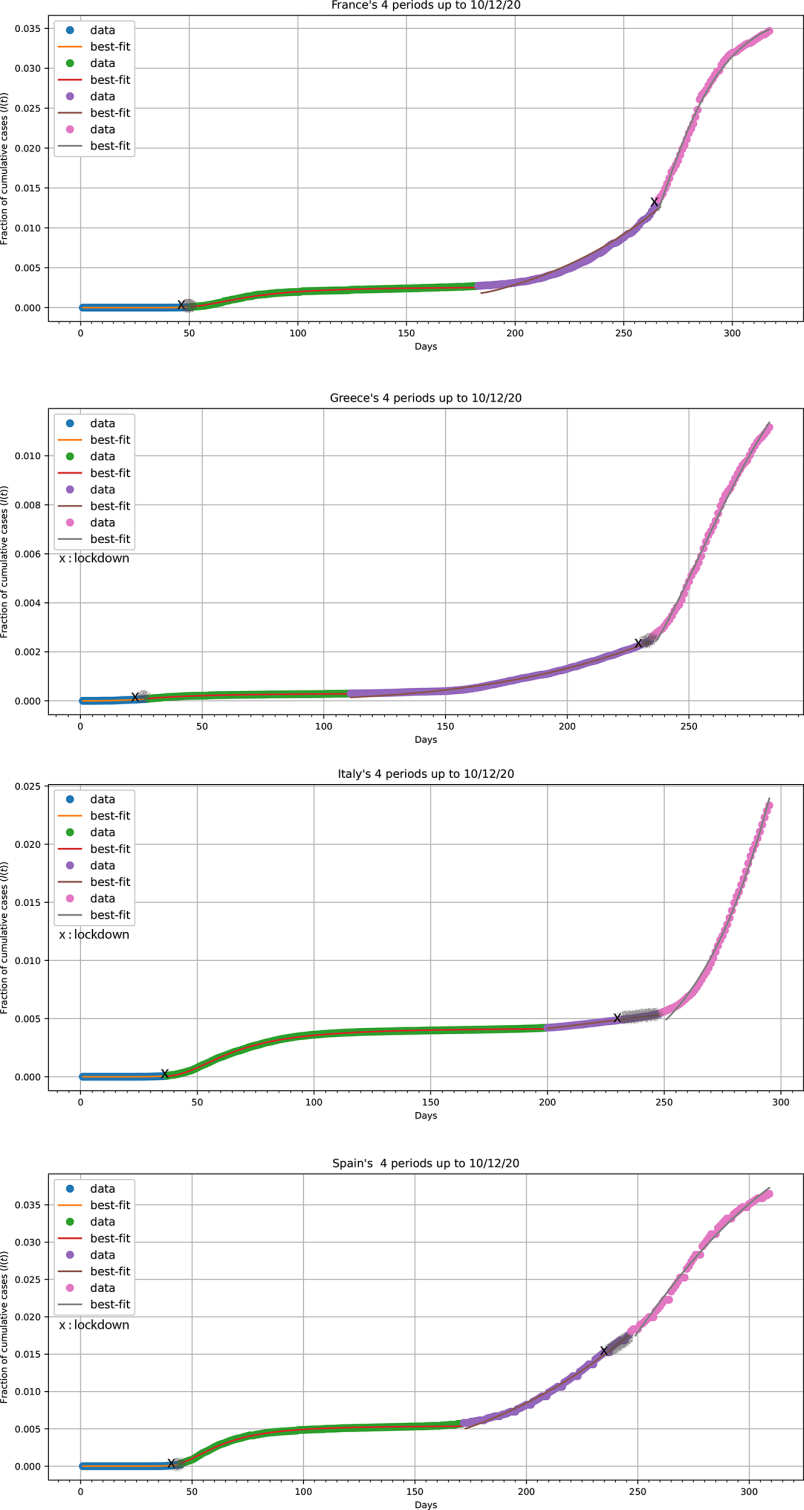
Best fits (solid lines) of Eqs. 6 and 4 for the herd kinetics periods and fractal kinetics periods, respectively, to data ^30^ (points) for France, Greece, Italy and Spain. The data correspond from time zero up to 10 December 2020. The gray line segments indicate fuzzy periods.

We focused on the data ^30^ of four model countries, namely, France, Greece, Italy, and Spain. Figure 4 shows for each one of the four countries, the fittings of Eqs. 6 and 4 to herd- and fractal-kinetics’ periods’ data, respectively. Parameter estimates derived are listed in Table 1. High *R ^2^* values listed in this Table indicate that the model of Eqs. 4 and 6, for all four countries, is in excellent agreement with the disease data except Italy’s fourth fractal kinetics’ period data.

**Table 1.** Estimates for the parameters of Eqs 6 and 4 derived from the fittings to herd kinetics periods and fractal kinetics periods data respectively, of France, Greece, Italy and Spain. ^30^ Estimates of the secondary parameters *t*_ip_, *t*_90%_, steady state infected fraction are also listed.

Parameters	France	Greece	Italy	Spain
*β*_herd1_	11.69 ± 0.09	2.38 ± 0.06	7.87 ± 0.13	11.09 ± 0.19
*α*_fractal 2_ (days) ^−l^	0.01 ± 4.89 × 10 ^−5^	0.02 ± 1.96 × 10 ^−4^	0.012 ± 1.69 × 10 ^−5^	0.01 ± 3.33 × 10 ^−5^
*h*_fractal 2_	4.71 ± 0.09	2.93 ± 0.06	4.39 ± 0.02	15.14 ± 0.06
*c*_fractal 2_	386.52 ± 2.56	3227 ± 30.19	238 ± 0.36	184 ± 0.54
*β*_herd 3_	5.08 ± 0.08	3.80 ± 0.04	1.17 ± 0.02	3.56 ± 0.04
*α*_fractal 4_ (days) ^−1^	0.003 ± l.348 × 10 ^−5^	0.003 ± 4.055 × 10 ^−5^	*high uncertainty value*	0.003 ± 5.62 × 10 ^−5^
*h*_fractal 4_	16.11 ± 0.54	9.01 ± 0.42	*high uncertainty value*	6.28 ± 0.43
*c*_fractal 4_	25.50 ± 0.29	53.58 ± 2.61	*high uncertainty value*	17.96 ± 0.94
*R*_1_^2 I^	0.999	0.994	0.997	0.996
*R*_2_^2 I^	0.990	0.993	0.999	0.996
*R*_3_^2 I^	0.984	0.991	0.992	0.993
*R*_4_^2 I^	0.995	0.996	0.995	0.991
*t*_ip_ *(estimated - observed)* (days) ^II^	17.6–18	24.4–25	*high uncertainty value*	21.5–23
*t*_90%_ (days) ^III^	31.9 ± 1.4	67.1 ± 4.2	*high uncertainty value*	63.6 ± 6.1
*I*( *t*→∞) ^IV^	0.038 ± 0.001	0.018 ± 0.001	*high uncertainty value*	0.053 ± 0.003

I: coefficient of determination.

II: estimated from numerical solution of second derivative.

III: estimated from equation 13.

IV: estimated from equation 5.

For the first herd kinetics’ period, the estimate for *β*_herd 1_ in Greece was found to be 2.38 ± 0.06, which is much smaller than for the other three countries. This is in agreement with the remarkably lower initial *I(t)* profile of Greece in Figure 4. We should emphasize the valid estimation of the parameter *β*_herd1_ for all countries studied. This is clear proof that the initial phase follows a power of time function ( Eq. 6) which is contrary to the general belief that the initial phase increases exponentially. This subexponential increase has been observed in the early phase of COVID-19 spreading in different parts of China. ^6^ During the first fractal kinetics’ period, the estimate for *α*_fractal 2_ in Greece was also higher, 0.02 ± 2 × 10 ^−4^ (days) ^−1^ compared with 0.010–0.012 (days) ^-1^ found for the other three countries. This leads to a shorter half-life of 42 days for Greece compared with an average of 63 days for the three other countries; this, coupled with the earlier lockdown rules imposed in Greece, explains the more rapid approach to the steady state ( Figure 4). The fractal exponent *h*_fractal 2_ was smaller in Greece, 2.93 ± 0.06, while for France, Italy and Spain it was 4.71 ± 0.09, 4.39 ± 0.02, 5.14 ± 0.06, respectively ( Table 1). On the contrary, the estimate for *c*_fractal 2_ in Greece 3227 ± 30.19 was roughly ten-times higher than in the other three countries, resulting in much lower *I*( *t*) steady-state value.

All countries remained in a slightly moving upwards quasi-steady state for 2–3 months ( Figure 4). This period was followed by a gradually increasing phase in the number of confirmed infected cases. Relaxed rules led to higher population mobility. All countries re-entered a herd kinetics’ period (blue concaving upwards segment in Figure 4). The estimates for *β*_herd 3_ were found 1.17 ± 0.02, 3.56 ± 0.04, 3.80 ± 0.04, 5.08 ± 0.08 for Italy, Spain, Greece and France, respectively, in full agreement with the visually increasing “curvature” of the blue concaving upwards segment of the four countries. All countries imposed preventive measures and lockdown rules several times ( Figure 4). For France, Greece and Spain a remarkably similar reliable estimate for *α*_fractal 4_, 0.003 (days) ^−1^ was found; this is indicative of a slow process with a half-life of 231 days. However, different *h*_fractal 4_ estimates, 16.11 *± *0.54 *,* 9.01 *±* 0.42 and 6.28 *±* 0.43 were found for France, Greece, and Spain *,* respectively. Assuming that the conditions will not change in the next time period, predictions, based on the parameter estimates of the fourth fractal kinetics period for the steady-state value and *t*_90%_, can be made for the three countries ( Table 1). On the contrary, the fitting of Eq. 4 to Italy’s fourth fractal kinetics’ period data was not equally successful and reliable parameters estimates for *h*_fractal 4_, *α*_fractal 4_ and *c*_fractal 4_ were not derived ( Table 1). This is due to the fact that the point of inflection has not been reached yet and therefore the fitting algorithm cannot converge to reliable parameters estimates.

The estimates for *t*_ip_ reported in Table 1 for France, Greece and Spain correspond to time (days) from the commencement of the fourth fractal kinetics’ period. These estimates were found to be in agreement with the observed values, which is an additional piece of evidence for the validity of the fractal model. An estimate for Italy’s *t*_ip_ was not obtained for reasons mentioned above. Besides, the fourth fractal kinetics period data were used to predict the *t*_90%_ (expressed in days from the commencement of this period) and the final steady-state (1/(1+ *c*)) for France, Greece and Spain ( Table 1).

### Analysis of COVID-19 data for countries deviating from the (HFF) ^2^ kinetic motif

**Figure 5.**
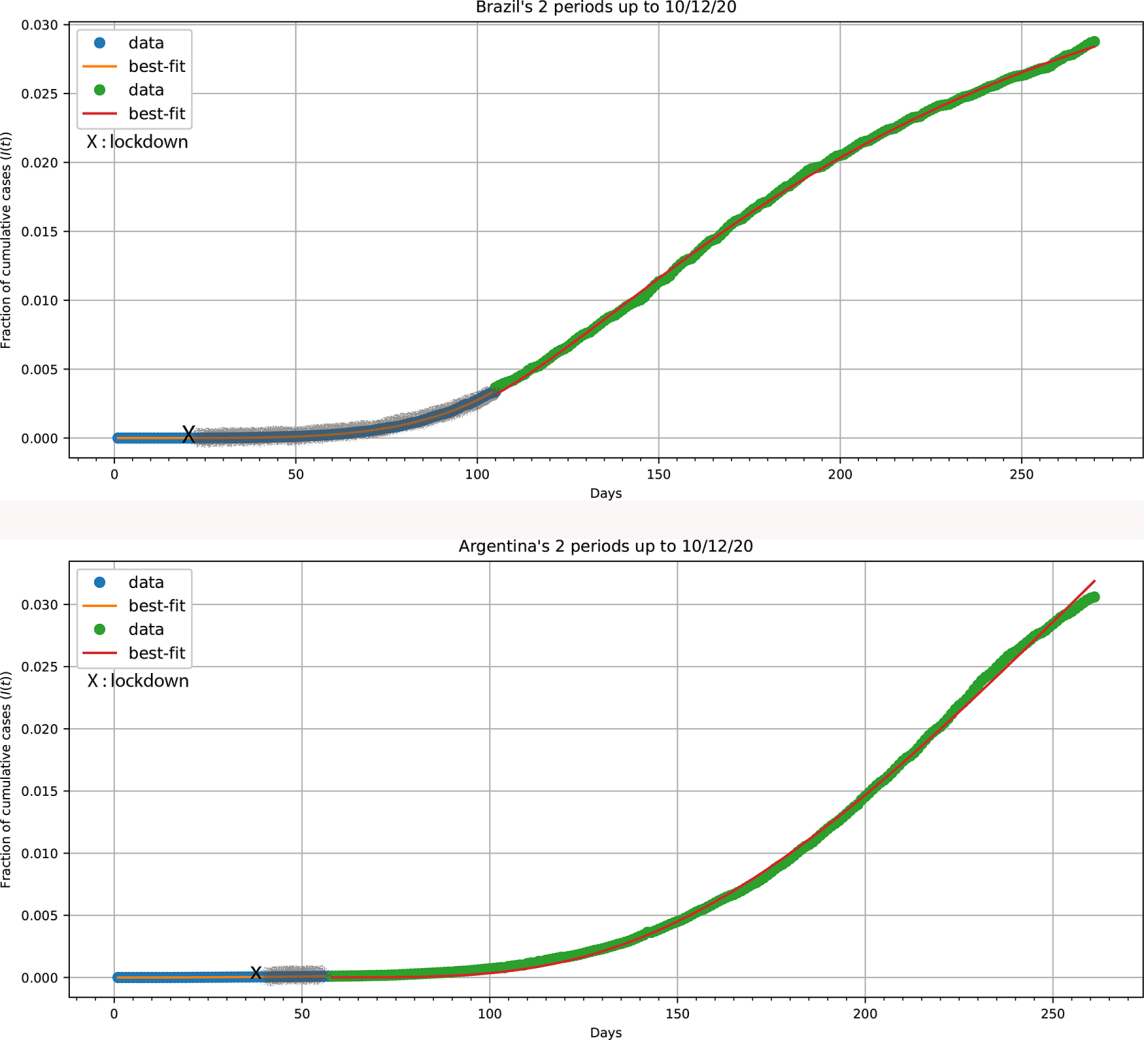
*I*( *t*) *versus* time plots for Argentina and Brazil. ^30^ Xs mark the implementation of mild preventive measures. The gray lines indicate the fuzzy period after the X point.

A large number of countries, besides the four analyzed, followed the (HFF) ^2^ kinetic motif shown in Figure 4, *e.g.*, Australia, China, Germany, Austria, United Kingdom. ^30^ Yet, several countries did not exhibit the (HFF) ^2^ motif, lacking a second wave and followed a “herd-fuzzy-fractal” (HFF) kinetic motif. Argentina and Brazil are examples of countries where strict/mild preventive measures were either not applied or did not work effectively. Both countries exhibit an (HFF) kinetic motif, Figure 5. Parameter values determined: Argentina, 1 ^st^ stage: ( *h* = 1), *β* = 1.929 ± 0.028; 2 ^nd^ stage: *h* = 2.189 ± 0.043, *α* = (1.754 ± 0.067) × 10 ^−3^ (days) ^−1^, *c* = 3.61 ± 0.38; Brazil, 1 ^st^ stage: ( *h* = 1), *β* = 4.633 ± 0.017; 2 ^nd^ stage: *h* = 2.892 ± 0.023, *α* = (4.044 ± 0.017) × 10 ^−3^ (days) ^−1^, *c* = 21.86 ± 0.24.

**Figure 6.**
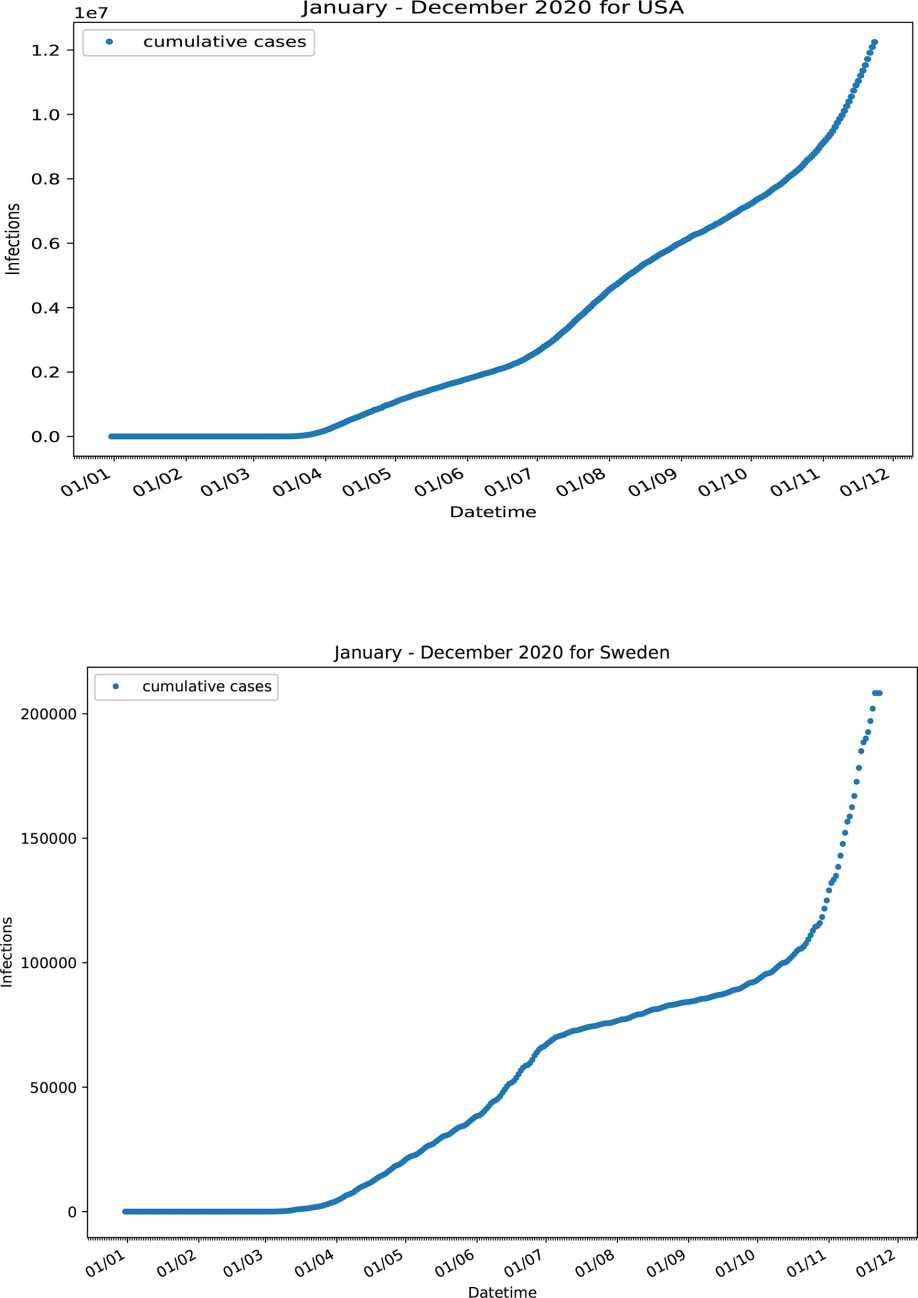
Confirmed infected cases for USA and Sweden. ^30^

On the other hand, some countries exhibited a more complex pattern, which deviates from the (HFF) ^2^ and (HFF) motifs. Infection data for USA did not follow either the (HFF) ^2^ or the (HFF) kinetic motif. The *I*( *t*) time profile never reached a steady state and the shape of the curve indicates a deformed three-wave like kinetic profile ( Figure 6). Probably both types of kinetics (herd and fractal) run concurrently for most of the time throughout the course of the pandemic, with the contribution of each varying with time. This is most likely due to different COVID-19 policy containment measures followed in different states around the country. Sweden intentionally applied the herd immunity strategy ^30^ during the COVID-19 pandemic. An initial herd-kinetics type continuous increase in the number of total infected cases reached a point of inflection around 20 July 2020, followed by a slower rate of increase of infected cases, ( Figure 6). Since neither strict measures nor lockdown rules were applied at that time, the shape of the curve should be attributed to a fractal kinetics-like self-organization of the society. A rather sharp increase after 10 September 2020 can be attributed to the increased mobility of the individuals since no relaxation measures were taken close to this date.

## Implications

The above results ( Figures 2, 3, Table 1) demonstrate that the fractal kinetics *SI* model is more akin to real life. Since the well-mixed hypothesis is the crux of the matter of the epidemiological models, ^5^ the use of not well-mixed hypothesis has important implications, which can metamorphosize airborne pandemics; these implications are discussed and itemized (designated with italics) below.

### The reproductive number

**Figure 7.**
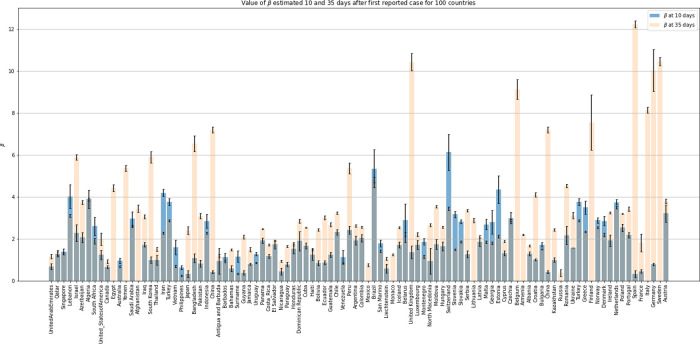
A bar plot of 100 countries based on the estimates with standard deviations for *β*, derived from the nonlinear regression analysis of data ^30^ using Eq. 6. Data of 10 and 35 days, after the first reported case, were analyzed. See also Table 2.

The reproductive number, *R*_0_ is not needed for the initial growth of the disease ^4^ being incompatible with the not well-mixed hypothesis, Figure 1. Limitations associated with the estimation of *R _0_*, can be found in numerous publications. Our results show that the time exponent *β* of Eq. 6 controls the time evolution of the disease throughout the initial herd kinetics’ period. In other words, *β* drives the initial phase of the disease spreading being the slope of Eq. 7, *i.e.* a linearized form of Eq. 6. The predominant role of *β* during the herd kinetics’ period can be also concluded from Eq. 12, which explicitly shows that the infected population fraction at the inflection point, *I*( *t*) *_ip_* is solely dependent on *β.* Although *R*_0_ and *β* are different, however, they can be used complementary to each other during the initial stages of the pandemics. Estimates for *β* derived from the analysis of herd kinetics’ period data at two time points from 100 countries are shown in Figure 7 and Table 2. The degree of uncertainty (standard deviation) for the estimates was found in most cases small; this was accompanied with high correlation coefficients (not shown). Overall, the estimates derived from the longer period of 35 days seem to be either similar or higher or significantly higher than these derived from the analysis of the shorter period (10 days) data. For some countries, the small number of confirmed infected cases in the first 10 days did not allow the estimation of *β.* In view of the diversity and variability of data presented in Figure 7, we quote the median values derived from the analysis of 100 countries, 2.44 (0.25–12.24) and 1.34 (0.20–6.13) for the *β* estimates corresponding to 35 and 10 days, respectively.

**Table 2.** *β* values and associated uncertainties ( *σ*) and fitting corresponding coefficients of determination ( *R*) derived from nonlinear regression analysis of data ^29^ from 100 countries using Eq. 6. Data of 10 and 35 days, after the first reported case, were analyzed. Part of these results is shown in Figure 7. Blanks are due to fragmented data that prevented the fitting procedure to converge.

#	Country	*β*_**35**_	*σ(β*_**35**_)	*N*	*R*	*β*_**10**_	*σ(β*_**10**_)	*N*	*R*
1	United Arab Emirates	1.154	0.107	36	0.99582	0.673	0.14517	11	0.979075
2	Qatar	1.329	0.126	32	0.99639	1.258	0.11773	8	0.988578
3	Singapore	1.294	0.054	36	0.99649	1.389	0.14623	11	0.992743
4	Lebanon	3.1	0.067	32	0.99914	4.012	0.56454	11	0.998925
5	Israel	5.891	0.136	33	0.99974	2.282	0.39644	10	0.99666
6	Azerbaijan	3.743	0.093	29	0.99935	2.071	0.22794	5	0.992274
7	Algeria	3.879	0.079	31	0.99942	3.894	0.44518	9	0.998676
8	South Africa	1.891	0.129	34	0.99804	2.605	0.43315	9	0.997136
9	United States of America	1.986	0.286	36	0.99825	1.24	0.22384	11	0.991278
10	Canada	0.92	0.094	36	0.99417	0.652	0.07839	11	0.978231
11	Egypt	4.418	0.169	32	0.99955			11	
12	Australia	0.683	0.043	36	0.99128	0.95	0.12042	11	0.986863
13	Yemen	5.373	0.144	36	0.99971			11	
14	Saudi Arabia	2.592	0.064	33	0.99884	2.955	0.34097	8	0.997505
15	Afghanistan	3.436	0.174	26	0.99919			7	
16	Iraq	3.048	0.093	34	0.99914	1.726	0.10908	11	0.994959
17	South Korea	5.9	0.27	36	0.99997	0.996	0.15335	11	0.987751
18	Thailand	1.5	0.089	36	0.99722	0.99	0.23089	11	0.987646
19	Iran	2.283	0.053	36	0.99862	4.198	0.17418	11	0.999016
20	Turkey	2.875	0.043	34	0.99905	3.765	0.1611	9	0.998585
21	Vietnam	0.692	0.065	36	0.99143	1.602	0.35587	11	0.994278
22	Philippines	0.247	0.034	33	0.97541	0.623	0.09151	11	0.977024
23	Japan	2.397	0.183	36	0.99874	0.32	0.15916	11	0.957422
24	Bangladesh	6.54	0.369	30	0.99978	1.084	0.21026	5	0.975912
25	Pakistan	3.092	0.118	31	0.99912	0.817	0.15819	7	0.975079
26	Indonesia	2.281	0.036	29	0.99843	2.848	0.32505	5	0.995896
27	India	7.2	0.15	36	0.99983	0.428	0.07002	11	0.966147
28	China	1.188	0.072	31	0.99562	0.942	0.60565	6	0.975897
29	Antigua and Barbuda	0.913	0.046	36	0.9941	1.137	0.18016	11	0.99
30	Barbados	1.487	0.049	34	0.99709	0.593	0.13826	9	0.971
31	Bahamas	0.334	0.04	31	0.97963	1.153	0.26721	6	0.982289
32	Suriname	2.095	0.093	34	0.99835	0.381	0.10594	9	0.956115
33	Guyana	1.489	0.109	35	0.99714	0.802	0.05252	10	0.981859
34	Jamaica	0.856	0.03	36	0.99355	1.275	0.07792	11	0.991663
35	Uruguay	2.468	0.029	36	0.9988	1.92	0.13438	11	0.995809
36	Panama	1.715	0.049	35	0.99773	1.168	0.09999	10	0.989569
37	Costa Rica	1.921	0.038	36	0.99815	1.735	0.21188	11	0.995005
38	El Salvador	0.928	0.046	36	0.99424	0.446	0.1619	11	0.967419
39	Nicaragua	1.641	0.052	32	0.99743	0.784	0.1117	7	0.973687
40	Paraguay	1.755	0.087	34	0.99778	1.525	0.22573	9	0.992582
41	Honduras	2.843	0.098	27	0.99889	1.894	0.46311	3	0.982547
42	Dominican Republic	2.541	0.032	33	0.9988	1.673	0.14523	8	0.992942
43	Cuba	1.5	0.058	36	0.99722	1.246	0.25306	11	0.991344
44	Haiti	2.43	0.052	35	0.99874	0.852	0.11752	10	0.983297
45	Bolivia	3.011	0.099	31	0.99908	0.864	0.0922	8	0.979807
46	Equador	2.675	0.088	36	0.99896	1.245	0.10828	11	0.991338
47	Guatemala	3.233	0.058	35	0.99924	2.324	0.12754	10	0.996768
48	Chile	0.831	0.039	36	0.99328	1.133	0.31607	11	0.989937
49	Venezuela	5.361	0.255	35	0.9997	2.416	0.186	10	0.996989
50	Peru	2.624	0.066	33	0.99886	1.92	0.20854	8	0.994462
51	Argentina	2.563	0.04	32	0.9988	2.036	0.17567	7	0.994346
52	Colombia	0.751	0.078	36	0.99229			11	
53	Mexico	4.814	0.125	36	0.99964	5.348	0.90313	11	0.999395
54	Brazil	1.42	0.053	35	0.99689	1.789	0.17175	10	0.994858
55	San Marino	1.051	0.072	30	0.99461	0.585	0.21517	5	0.94762
56	Liechtenstein	1.24	0.046	24	0.99516			3	
57	Monaco	2.539	0.057	36	0.99885	1.717	0.14569	11	0.994915
58	Iceland	2.065	0.239	28	0.9981	2.904	0.76791	6	0.996674
59	Belarus	10.44	0.415	36	0.99992	1.371	0.30641	11	0.992587
60	United Kingdom	2.212	0.099	29	0.99834	1.707	0.21415	5	0.988902
61	Luxembourg	1.148	0.064	36	0.99578	1.867	0.15733	11	0.995599
62	Montenegro	2.667	0.071	27	0.99876	0.942	0.60567	6	0.975901
63	North Macedonia	3.538	0.065	34	0.99935	1.743	0.2317	9	0.994102
64	Moldova	2.556	0.048	35	0.99885	1.643	0.21845	10	0.994054
65	Hungary	3.438	0.086	36	0.99933	6.138	0.86169	11	0.999545
66	Switzerland	1.491	0.031	35	0.99715	3.169	0.15303	10	0.998181
67	Slovenia	1.853	0.045	35	0.998	2.822	0.08282	10	0.997738
68	Slovakia	3.358	0.057	33	0.99927	1.264	0.16136	8	0.988674
69	Serbia	2.887	0.105	27	0.99892			4	
70	Lithuania	2.102	0.066	32	0.9983	1.845	0.18954	7	0.993247
71	Latvia	1.855	0.059	36	0.99804	2.688	0.25161	11	0.997706
72	Malta	1.799	0.056	33	0.99783	2.798	0.57651	8	0.997231
73	Georgia	2.114	0.074	31	0.99828	4.362	0.66006	7	0.998736
74	Estonia	1.882	0.046	34	0.99802	1.304	0.11802	9	0.990376
75	Cyprus	2.884	0.066	36	0.99909	2.992	0.27071	11	0.998119
76	Czechia	9.135	0.462	36	0.9999			11	
77	Belgium	2.194	0.037	27	0.99825			2	
78	Armenia	1.667	0.042	36	0.99766	1.28	0.09468	11	0.991712
79	Albania	4.1	0.112	33	0.99949	1.013	0.04827	10	0.987014
80	Croatia	1.497	0.027	34	0.99712	1.725	0.10106	9	0.993996
81	Bulgaria	7.2	0.15	36	0.99983	0.428	0.07002	11	0.966147
82	China	2.432	0.051	36	0.99877	0.995	0.1101	11	0.987732
83	Kazakhstan	0.375	0.161	36	0.98316			11	
84	Russia	4.531	0.075	34	0.99959	2.166	0.43266	9	0.995986
85	Romania	3.111	0.149	26	0.99903			2	
86	Ukraine	2.875	0.043	34	0.99905	3.765	0.1611	9	0.998585
87	Turkey	2.346	0.034	34	0.99864	3.493	0.3283	9	0.998361
88	Greece	7.563	1.321	34	0.99984			11	
89	Finland	2.532	0.049	36	0.99885	2.888	0.1294	11	0.997991
90	Norway	2.191	0.082	36	0.99852	2.857	0.21442	11	0.99795
91	Denmark	3.244	0.054	34	0.99923	1.916	0.26743	9	0.995004
92	Ireland	3.5	0.061	36	0.99935	3.75	0.12038	11	0.998774
93	Netherlands	3.192	0.02	34	0.99921	2.533	0.17424	9	0.996984
94	Poland	3.418	0.098	36	0.99933	2.176	0.13686	11	0.99664
95	Portugal	12.24	0.159	36	0.99994	0.32	0.15915	11	0.957416
96	Spain	1.804	0.419	36	0.99795	0.454	0.077	11	0.967925
97	France	8.139	0.145	36	0.99987			11	
98	Italy	10.04	0.991	36	0.99991	0.788	0.06693	11	0.982925
99	Germany	10.46	0.196	36	0.99992			11	
100	Sweden	3.799	0.114	36	0.99944	3.211	0.41481	11	0.998352

### Exponential *versus* power growth

The classical phraseology “ *the exponential growth of the disease*” used by medical doctors, scientists and laymen is questionable. This phrase is related to the approximate solution of the *SIR* model, which is an exponential function, when the parameter of the recovery rate is equal to zero. ^5^ Based on our theoretical results and the good fittings of Eq. 6 to data of herd kinetics’ period ( Figure 4), *“the herd kinetics’ period seems to obey a power of time function”.* According to Eq. 2, *β* drives the disease spreading when *h* = 1 and the rate of infection is inversely proportional to time. This is in agreement with the real-life conditions because of the continuous reduction of the probability of infection as a function of time ( *β*/ *t*). However, the resemblance of the *I*( *t*) profiles of the classical, *h* = 0 and the special case *h* = 1 in Figure 2 makes the discernment of the kinetics of the initial phase difficult.

*Herd immunity*

Herd immunity ^5^ calculations rely on an estimate for *R*_0_ and syllogisms based on the relative magnitude, *λ* = *R*( *t*)/ *R*_0_, which is the proportion of the population that is susceptible to catching the disease. If preventive measures are not applied, an estimate for the time needed to reach a certain level of the infected population fraction, e.g., *I*( *t*) *= *0.6 ensuring herd immunity can be obtained from Eq. 7. Assuming an infected individual at time *t*=1, i.e., $\frac{1}{I\left(t=1\right)}=N$ , where *N* is the population of the country, then, from Eq. 7, we get *c* = *N* − 1 ≈ *N.* Hence, the time *t*_hi_ needed to reach a certain level of herd immunity *I* ( *t*_hi_) under non preventive measures is

$$t_{\mathrm{hi}}={\left(\frac{N}{\frac{1}{I\left(t_{\mathrm{hi}}\right)}-1}\right)}^{\frac{1}{\beta }}$$ (14)

**Figure 8.**
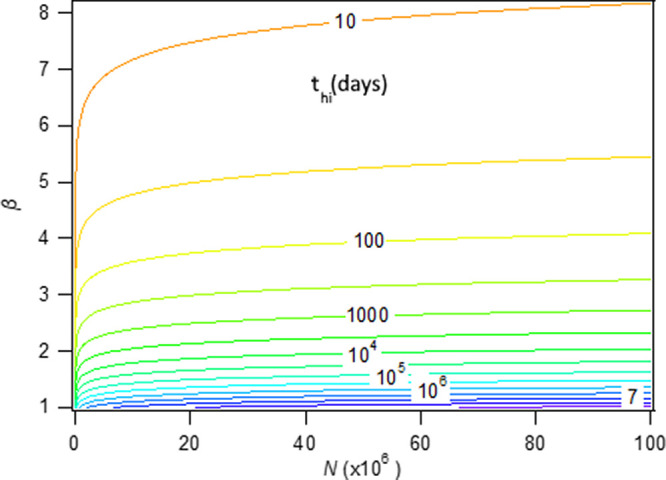
A contour plot based on Eq. 14 showing the time required to reach herd immunity level *I* ( *t*_hi_) = 0.6 for various values of parameter *β* and population size *N.*

Figure 8 shows *t*_hi_ as a function of *β* and *N* assigning *I* ( *t*_hi_) = 0.6. It can be seen that population size has a mild effect, whereas the apparent transmissibility constant *β* severely reduces *t*_hi_. Eq. 14 can be used at the initial stages of the pandemics and requires only a valid estimate for *β.* This will certainly provide valuable information for authorities, if coupled with estimates of the mortality rate and deaths, prior to a decision for a herd-immunity policy. ^33^ Caution should be exercised with the use of Eq. 14, since it can be applied only under the strict assumption of herd kinetics operating throughout the entire period of the disease spreading. The example of Sweden ( Figure 6) shows that societies can exhibit self-organization and move to a fractal kinetics’ mode.

*Deviation from the herd kinetic profile after the imposition of lockdown*

**Figure 9.**
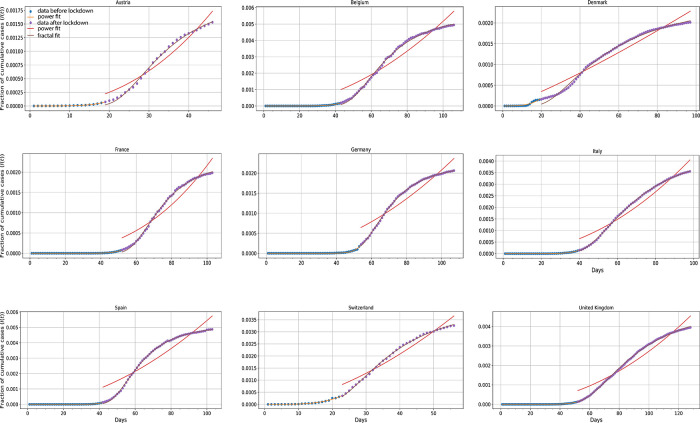
*I*( *t*) *versus* time plots for Austria, Belgium, Denmark, France, Germany, Italy, Spain, Switzerland, United Kingdom. ^30^ The blue dots represent cumulative infected cases up to lockdown datum points. ^16^ The orange lines depict the power fit to these data. Purple dots represent data after lockdown imposition whereas the purple lines are their superimposed fractal fits. Red lines depict the hypothetical power fit to the aforementioned data points in the event that Covid-19 propagation followed a power law pattern. *R* ^2^ values for all nine countries were measured higher than 0.98.

Cumulative data of infected people from nine countries (Austria, Belgium, Denmark, France, Germany, Italy, Spain, Switzerland, United Kingdom) were gathered and analyzed under two different prisms. Analysis was broken down into two parts, before and after imposition of strict preventive measures (lockdown) ( Figure 9). For the first period, the herd kinetic motif where *h* = 1 ( Eq. 6) was found to be adequate, whereas after lockdown clearly fails. The latter period was also analyzed using the fractal kinetic motif of *h* > 1 with very persuasive goodness of fit ( Figure 9). In all cases, *R* ^2^ was greater than 0.98. This pictorial divergence shows that after implementing mobility restrictions the evolution of the pandemic could not be captured by a power law expression, but rather by a fractal kinetic one ( Eq. 4) which eventually leads to a plateau of cumulative cases.

*Model predictions*

According to Jewell *et al*., ^1^ the ability of current models to predict is very poor. Our work demonstrates that the herd kinetics’ period is described by Eq. 6, while the kinetic motif “herd-fuzzy-fractal” should be taken into account in the modeling work. Apparently, these approaches have not been implemented so far. Roughly, predictions during the herd kinetics’ period can be based on a valid estimate for *β*, Eq. 6. Under preventive measures, valid estimates for the parameters of the model ( Eq. 4, *h*>1) can be derived and used for predictive purposes provided that data beyond the point of inflection are available (see Table 1).

## Conclusions

Since the early days of epidemics’ modeling, ^3^ a great deal of work has been done and now there is a change of paradigm. Interestingly, the results of our work are in full agreement with the basic conclusion of the most recent, extensive and elegant COVID-19 study ^16^ based on the effective reproduction number *R*( *t*), “… that major non-pharmaceutical interventions—and lockdowns in particular—have had a *large effect* on reducing transmission”. Our approach quantifies this large effect on the basis of Eq. 4, which captures the dynamics of the disease under “herd kinetics’” and “fractal kinetics’” conditions. In addition, our herd kinetics’ period results are in full agreement with the observations of the distinctive subexponential increase of confirmed cases during the early phase of the epidemic in China, contrasting an initial exponential growth expected for an unconstrained outbreak. ^6^ The present fractal *SI* model can be extended to its *SIR* analogue, with the caveat that the corresponding differential equations require numerical solution. In conclusion, the fractal kinetics *SI* model with the kinetically established herd period as well as the (HFF) ^2^ or (HFF) kinetic motifs opens up a new era in the field of epidemiological models for airborne pandemics.

## Data Availability

The underlying data are publicly available from https://www.ecdc.europa.eu/en/covid-19/data. ^30^

## Software availability

Archived source code available from: https://doi.org/10.5281/zenodo.4940276. ^31^

Data are available under the terms of the Creative Commons Attribution 4.0 International license (CC-BY 4.0).
