# Supplementary material for: Interpreting airborne pandemics spreading using fractal kinetics’ principles
Source: F1000Res. 2021 Jul 20;10:609. [Version 1] doi: 10.12688/f1000research.53196.1 (PMC8686328; doi:10.12688/f1000research.53196.1)
Supplement: Peer review history

# Peer review: Reviewer response for version 1

**DOI:** 10.5256/f1000research.56555.r100029

**Published:** 2021-12-16

**Authors:** Juan J Nieto

**Affiliations:** 1 Institute of Mathematics, Universidade de Santiago de Compostela, Santiago de Compostela, 15782, Spain

- This is a timely and interesting contribution to the study of the dynamics of the COVID-19. Since some models do not capture well the dynamics, it is considered a fractional principle.
- Fractal and fractional models have considered, for example in Ndaïrou *et al. *(2021 ^1^) and Bushnaq *et al. *(2021 ^2^). 
- I would recommend to continue this approach with updated data.

Is the work clearly and accurately presented and does it cite the current literature?

Partly

If applicable, is the statistical analysis and its interpretation appropriate?

Yes

Are all the source data underlying the results available to ensure full reproducibility?

Yes

Is the study design appropriate and is the work technically sound?

Yes

Are the conclusions drawn adequately supported by the results?

Yes

Are sufficient details of methods and analysis provided to allow replication by others?

Yes

Reviewer Expertise:

Pure and Applied Mathematics, Biomathematics

I confirm that I have read this submission and believe that I have an appropriate level of expertise to confirm that it is of an acceptable scientific standard.

## References

[1] : Fractional model of COVID-19 applied to Galicia, Spain and Portugal. *Chaos Solitons Fractals* .2021;144: 10.1016/j.chaos.2021.110652 110652 10.1016/j.chaos.2021.110652 33519122PMC7833683

[2] : Control of COVID-19 dynamics through a fractional-order model. *Alexandria Engineering Journal* .2021;60(4) : 10.1016/j.aej.2021.02.022 3587-3592 10.1016/j.aej.2021.02.022

# Peer review: Reviewer response for version 1

**DOI:** 10.5256/f1000research.56555.r100027

**Published:** 2021-12-07

**Authors:** Jinjun Zhang

**Affiliations:** 1 Beijing Emergency Medicine Research Institute, Beijing, China

Thanks for the opportunity to review this manuscript. This article by Panos Macheras, Athanasios A. Tsekouras and Pavlos Chryssafidis gives a consistent analysis for a not well-mixed system using fractal kinetic principles. They derived a three arameter model and show that the fractal exponent of time larger than unity can capture the impact of preventive measures affecting population mobility, and lead to the conclusion that the fractal kinetics model can be used as a prototype for the analysis of all contagious airborne pandemics. Overall, The study is well-designed and data analyses are well performed.

Is the work clearly and accurately presented and does it cite the current literature?

Yes

If applicable, is the statistical analysis and its interpretation appropriate?

I cannot comment. A qualified statistician is required.

Are all the source data underlying the results available to ensure full reproducibility?

Yes

Is the study design appropriate and is the work technically sound?

Yes

Are the conclusions drawn adequately supported by the results?

Yes

Are sufficient details of methods and analysis provided to allow replication by others?

Yes

Reviewer Expertise:

NA

I confirm that I have read this submission and believe that I have an appropriate level of expertise to confirm that it is of an acceptable scientific standard.

# Peer review: Reviewer response for version 1

**DOI:** 10.5256/f1000research.56555.r93405

**Published:** 2021-09-22

**Authors:** Raoul Kopelman

**Affiliations:** 1 Department of Chemistry, University of Michigan, Ann Arbor, MI, USA

Overall this is an important contribution, pointing out the occasionally fallacious assumption in epidemiological theories of a “well stirred” population. While in many chemical reactions “well stirred” conditions are met, or taken care of to be met, the adoption in the standard epidemiology approaches of a similar condition is questionable. While it may be a good approximation for an animal model, or for a primitive society where no precautions against infection are practiced, this is questionable for a modern society, where isolation of the sick and preventive measures by the healthy, e.g., wearing masks, are a standard, or even mandated *modus operandi*. This justifies rejecting the “classical reaction kinetics” model and replacing it by a “non-classical reaction kinetics” model, which was originally called “fractal reaction kinetics” (Ref 8).

 While with the new model more adjustable parameters are introduced, which should be emphasized by the authors, the agreement with the spread of Covid in a large number of countries is impressive. So is the agreement of the classical limit (“h=0”) with the spread of the pandemic in Sweden, where no preventive restrictions were adopted, with the aim of reaching “herd immunity” (an animal model).

**Minor Comments:**

1. Explain better the definition of the last factor in Eq. 1.
2. Explain better the definition of the last factor in Eq. 2. Also discuss the positive vs. negative values of h.
3. Straighten out and explain eq. 3.
4. Carefully define “alpha” in eq. 4.
5. In Eq. 6, explain wherefrom comes “11”.
6. Eq. 8, ditto.
7. Eq. 10, ditto.
8. Eq. 12, explain wherefrom comes “12”.
9. Eq. 13, explain wherefrom comes “11”.
10. Clarify eq. 14.

Is the work clearly and accurately presented and does it cite the current literature?

Yes

If applicable, is the statistical analysis and its interpretation appropriate?

Yes

Are all the source data underlying the results available to ensure full reproducibility?

Yes

Is the study design appropriate and is the work technically sound?

Yes

Are the conclusions drawn adequately supported by the results?

Partly

Are sufficient details of methods and analysis provided to allow replication by others?

Yes

Reviewer Expertise:

NA

I confirm that I have read this submission and believe that I have an appropriate level of expertise to confirm that it is of an acceptable scientific standard, however I have significant reservations, as outlined above.

# Response

**Published:** 2021-10-18

**Authors:** Pavlos Chrys

Response to comments by R. Copelman.

 We thank the reviewer for his constructive critique. Regarding the minor comments and questions, we have the following replies to each point raised.

- *Explain better the definition of the last factor in Eq. 1.*
- **Response:** This equation is presented in detail in Ref. 11.
- *Explain better the definition of the last factor in Eq. 2. Also, discuss the positive vs. negative values of h.*
- **Response: **[1- *I*( *t*)] is the fraction of the susceptible population. Negative *h* values don't come up in actual data.
- *Straighten out and explain eq. 3.*
- **Response:** Eq. 3 is described in Ref. 7.
- *Carefully define “alpha” in eq. 4.*
- **Response: **See line above Eq. 4.
- *In Eq. 6, explain wherefrom comes “11”.*
- **Response: **What does "11" stand for? There is no "11" in Eq. 6. Eq. 6 is derived from Eq. 3 by taking the limit *h*->1 and implementing L'Hopital's rule, i.e., taking the derivative of numerator and denominator in the exponent and then taking the limit. We will clarify the point in the next version of the text.
- *Eq. 8, ditto.*
- **Response:** Eq. 8 is derived from Eq. 7 after doing some straightforward algebra.
- *Eq. 10, ditto.*
- **Response: **The derivation of Eq. 10 is described right before Eq. 9 and repeated before Eq. 11.
- *Eq. 12, explain wherefrom comes “12”.*
- **Response:** Eq. 12 is derived from Eq. 6 following the procedure described before Eq. 9.
- *Eq. 13, explain wherefrom comes “11”.*
- **Response:** Eq. 13 is derived from Eq. 4 by setting the right-hand side of the latter equal to 0.9 times *I*( *t*->infinity) and solving for *t*.
- *Clarify eq. 14.*
- **Response: **Eq. 14 tells us when the fraction of infected individuals will reach a certain level given the size of the population and exponent *β*. Here again, we will add a brief explanation in the updated version of the text.
